# The Arabidopsis Lectin Receptor Kinase LecRK-V.5 Represses Stomatal Immunity Induced by *Pseudomonas syringae* pv. *tomato* DC3000

**DOI:** 10.1371/journal.ppat.1002513

**Published:** 2012-02-09

**Authors:** Marie Desclos-Theveniau, Dominique Arnaud, Ting-Yu Huang, Grace Jui-Chih Lin, Wei-Yen Chen, Yi-Chia Lin, Laurent Zimmerli

**Affiliations:** Department of Life Science and Institute of Plant Biology, National Taiwan University, Taipei, Taiwan; Michigan State University, United States of America

## Abstract

Stomata play an important role in plant innate immunity by limiting pathogen entry into leaves but molecular mechanisms regulating stomatal closure upon pathogen perception are not well understood. Here we show that the *Arabidopsis thaliana* L-type lectin receptor kinase-V.5 (LecRK-V.5) negatively regulates stomatal immunity. Loss of *LecRK-V.5* function increased resistance to surface inoculation with virulent bacteria *Pseudomonas syringae* pv *tomato* DC3000. Levels of resistance were not affected after infiltration-inoculation, suggesting that LecRK-V.5 functions at an early defense stage. By contrast, lines overexpressing *LecRK-V.5* were more susceptible to *Pst* DC3000. Enhanced resistance in *lecrk-V.5* mutants was correlated with constitutive stomatal closure, while increased susceptibility phenotypes in overexpression lines were associated with early stomatal reopening. Lines overexpressing *LecRK-V.5* also demonstrated a defective stomatal closure after pathogen-associated molecular pattern (PAMP) treatments. *LecRK-V.5* is rapidly expressed in stomatal guard cells after bacterial inoculation or treatment with the bacterial PAMP flagellin. In addition, *lecrk-V.5* mutants guard cells exhibited constitutive accumulation of reactive oxygen species (ROS) and inhibition of ROS production opened stomata of *lecrk-V.5*. LecRK-V.5 is also shown to interfere with abscisic acid-mediated stomatal closure signaling upstream of ROS production. These results provide genetic evidences that LecRK-V.5 negatively regulates stomatal immunity upstream of ROS biosynthesis. Our data reveal that plants have evolved mechanisms to reverse bacteria-mediated stomatal closure to prevent long-term effect on CO_2_ uptake and photosynthesis.

## Author Summary

During their lifetime, plants face numerous pathogenic microbes. Plants recognize microbial pathogens via plant receptors and recognition leads to the activation of a general defense response. Some foliar pathogens such as bacteria enter plant leaves through natural surface openings such as stomata. To restrict bacterial entry, plants close stomata upon contact with bacteria. A better understanding of stomatal immunity may lead to development of crops with improved disease resistance. Here, we used the model plant *Arabidopsis thaliana* to study activation of defense responses after infection by *Pseudomonas syringae* pv. *tomato* (*Pst*) DC3000 bacteria. We found that a gene not previously known to function in the defense response, *LecRK-V.5*, is modulating Arabidopsis resistance. By studying plants with mutations in or overexpressing this gene, we show that *LecRK-V.5* negatively regulates plant stomatal immunity to *Pst* DC3000. In addition, *LecRK-V.5* is rapidly expressed at stomata upon activation of the general defense response. Plants with mutations in *LecRK-V.5* also demonstrated constitutive accumulation of reactive oxygen species in stomatal guard cells. We conclude that LecRK-V.5 is a protein that negatively regulates closure of stomata upon bacterial infection.

## Introduction

Plants are continuously exposed to a variety of microorganisms and have elaborated defense mechanisms to successfully avoid infection by limiting pathogen invasion and multiplication. The earliest event in plant defense response is recognition of microbial molecular signatures called pathogen- or microbe-associated molecular patterns (PAMPs or MAMPs) by pattern recognition receptors (PRRs) located at the plasma membrane [1]. One of the best characterized PRR is the *Arabidopsis thaliana* receptor kinase Flagellin Insensitive 2 (FLS2) that recognizes and interacts with the peptide flg22, the biologically active epitope of the bacterial PAMP flagellin. PAMPs perception initiates a variety of basal defense response referred to as PAMP-triggered immunity (PTI), which mostly includes reactive oxygen species (ROS) production, increase in Ca^2+^ influx, activation of mitogen-activated protein kinase (MAPK) cascades, transcriptional activation, callose deposition and stomatal closure [2].

Stomata are microscopic pores surrounded by a pair of guard cells and located at the leaf epidermis. They control CO_2_ uptake for photosynthesis, water loss during transpiration and play a crucial role in biotic and abiotic stress tolerance [3]. Stomata are critical during the plant innate immune response [4], [5]. Bacteria such as *Pseudomonas syringae* pv *tomato* (*Pst*) strain DC3000 induce stomatal closure in Arabidopsis within 1 to 2 h post inoculation. However, *Pst* DC3000 is able to reopen stomata 3 to 4 h after infection through the action of coronatine (COR) [4]. COR acts downstream of ROS accumulation and reverses the inhibitory effects of flg22 on both K^+^ (in) currents and stomatal opening [6]. Both salicylic acid (SA) and abscisic acid (ABA) synthesis and signaling pathways are required during bacterial- and PAMP-induced stomatal closure in Arabidopsis [4], [7]. Several studies suggest that PAMP-induced stomatal closure share common signaling pathway with the ABA-induced stomatal closure [4], [6], [8]. PAMP-induced stomatal closure requires the synthesis of H_2_O_2_ [9]. In Arabidopsis, ABA- and flg22-induced ROS is dependent on the NADPH oxidase Rboh [10], [11], [12] and *Rboh* is specifically required for flg22- and bacterial-induced stomatal closure [13].

The Arabidopis lectin receptor kinase *LecRK-V.5* (also known as *LecRK1* or *LecRK-a1*) belongs to a multigenic family comprising 45 members [14], [15], [16]. LecRK-V.5 protein is likely localized at the plasma membrane and the kinase domain can be phosphorylated on serine residues [14], [17]. *LecRK-V.5* is up-regulated in senescing leaves, and is induced by wounding and oligogalacturonic acid treatments [18]. Although a significant number of *LecRKs* demonstrate an increased expression upon pathogen or elicitor treatments [16], only a few reports demonstrated a function for a LecRK in plant-pathogen interactions [19], [20], [21]. In this study, we show that *lecrk-V.5* is a key regulatory gene in stomatal immunity. In particular, our data suggest that LecRK-V.5 negatively regulates bacterial- and PAMP-triggered stomatal closure upstream of ROS production.

## Results

### LecRK-V.5 negatively regulates disease resistance to bacteria

**Figure 1:**
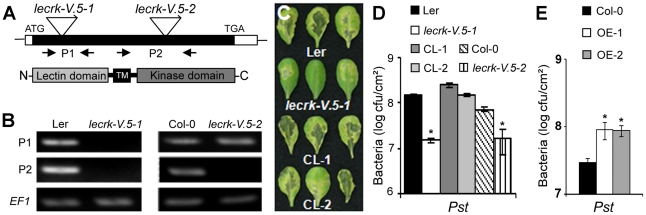
LecRK-V.5 negatively regulates disease resistance to bacteria. (A) Insertional mutation sites in two *lecrk-V.5* mutant lines. Ds transposon (*lecrk-V.5-1*) and T-DNA (*lecrk-V.5-2*) insertion sites are shown. Filled box represents exon and arrows denote the different positions of the primers used in the RT-PCR experiments in (B). The relative position of lectin, transmembrane (TM) and kinase domains in LecRK-V.5 predicted protein structure is indicated. (B) RT-PCR analysis of *LecRK-V.5* transcripts in WT and *lecrk-V.5* mutants. *EF-1* was used as a control. (C) Disease symptoms assessed 3 days after dip-inoculation with 1×10^7^ cfu.ml^−1^ *Pst* DC3000 in WT (Ler), *lecrk-V.5-1* and two complemented lines (CL-1 and CL-2). (D) Bacterial growth at 3 days post-inoculation (1×10^7^ cfu.ml^−1^ *Pst* DC3000 (*Pst*)) in WT (Col-0 and Ler), *lecrk-V.5* mutants and two complemented lines (CL-1 and CL-2). (E) Bacterial titers evaluated 3 days after dip-inoculation with 1×10^7^ cfu.ml^−1^ *Pst* DC3000 (*Pst*) in lines overexpressing *LecRK-V.5* (OE-1 and OE-2). For (D) and (E), data represent average ± SD. Statistical differences between WT controls and mutants or transgenics are detected with a *t* test (*P*<0.01, n = 6). All experiments were repeated at least three times with similar results.

To identify novel players in the Arabidopsis defense response, a reverse genetic approach was undertaken with the PAMP- and bacteria-responsive *LEGUME-LIKE LECTIN RECEPTOR KINASE-V.5* (*LecRK-V.5*, At3g59700) [16]. Towards this goal, we isolated *lecrk-V.5-1*, a transcriptional knockout *Ds* transposon insertion line and *lecrk-V.5-2*, a T-DNA insertion line producing a truncated transcript (Figure 1A, B). Arabidopsis mutants were dip-inoculated with the virulent bacteria *Pst* DC3000 and disease progression was evaluated. *lecrk-V.5-1* and *lecrk-V.5-2* mutants developed less disease symptoms and lower bacterial titers than wild-type (WT) controls (Figure 1C, D). To confirm that the mutation in *LecRK-V.5* is responsible for the enhanced *Pst* DC3000 resistance observed in the *lecrk-V.5-1* mutant, *35S::LecRK-V.5* (CL-1 for complemented line 1) and *Pro_LecRK-V.5_*::*LecRK-V.5-HA* (CL-2 for complemented line 2) constructs were produced for complementation analysis. *LecRK-V.5* was up-regulated about 75 times in CL-1 while CL-2 showed a WT level of *LecRK-V.5* expression (Figure S1). The mutant *lecrk-V.5-1* transformed with both constructs demonstrated WT susceptibility to *Pst* DC3000 dip-inoculation (Figure 1C, D). To further ascertain whether LecRK-V.5 is involved in bacterial resistance, we generated transgenic Arabidopsis Col-0 plants harboring the *35S::LecRK-V.5* (OE-1) and *35S::HA-LecRK-V.5* (OE-2) constructs. Both lines demonstrated a strong up-regulation of *LecRK-V.5* characterized by expression levels about 250 times higher than WT (Figure S1). Such overexpression lines demonstrated higher *Pst* DC3000 titer levels than WT controls (Figure 1E). Collectively, these data indicate that LecRK-V.5 negatively regulates Arabidopsis resistance to *Pst* DC3000.

### Phenotypes of *lecrk-V.5* mutants and lines over-expressing *LecRK-V.5* are associated with stomatal immunity

**Figure 2:**
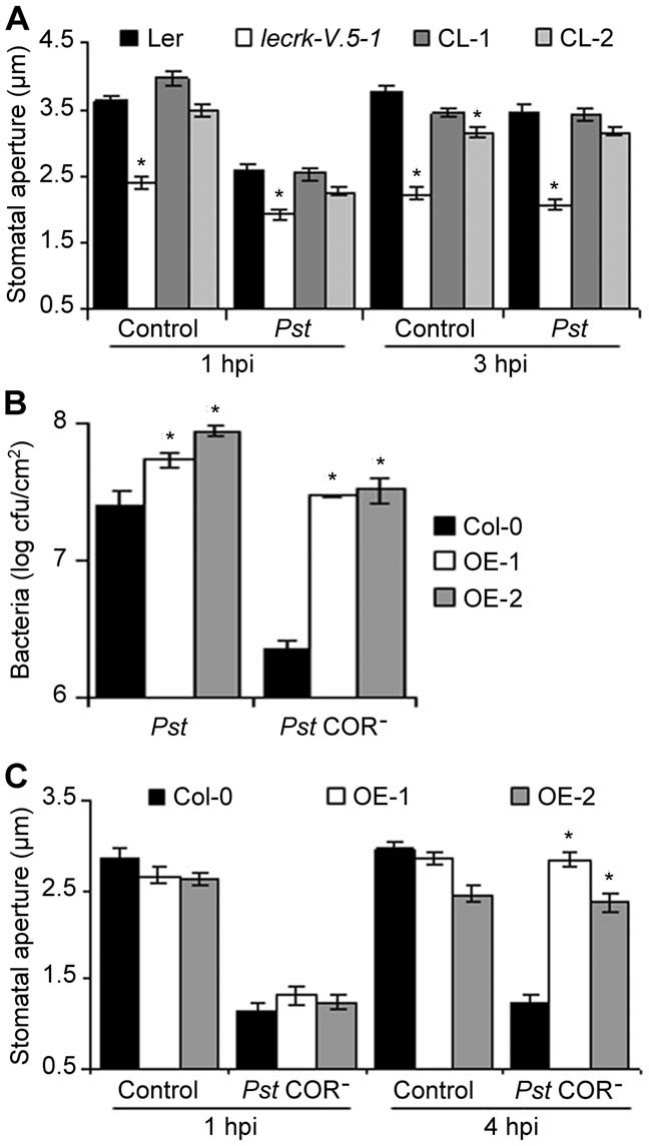
Altered stomatal immunity responses in *lecrk-V.5-1* and in lines over-expressing *LecRK-V.5*. (A) Stomatal apertures in epidermal peels exposed to MES buffer (control) or 1×10^8^ cfu.ml^−1^ *Pst* DC3000 (*Pst*) for 1 or 3 hrs. (B) Bacterial growth assessed 3 days after dip-inoculation with 1×10^7^ cfu.ml^−1^ *Pst* DC3000 (*Pst*) or *Pst* DC3000 COR^−^ (*Pst* COR^−^) in WT Col-0 and overexpression lines (OE-1 and OE-2). Values are the means ± SD. Statistical differences between WT controls and OE lines are detected with a *t* test (*P*<0.01, n = 6). (C) Stomatal aperture in WT Col-0 and overexpression lines (OE-1 and OE-2) after 1 and 4 hours incubation in MES buffer (Control) or 1×10^8^ cfu.ml^−1^ *Pst* DC3000 COR^−^ (*Pst* COR^−^). For (A) and (C), results are shown as mean of ≥60 stomata measurements ± SE. Asterisks indicate significant differences between WT and mutant/CL/OE lines based on a *t* test (*P*<0.001). hpi, hour post inoculation. All experiments were repeated at least three times with similar results.

Although *lecrk-V.5-1* and *lecrk-V.5-2* were more resistant to *Pst* DC3000 after dip-inoculation (Figure 1C, D), both mutants demonstrated WT susceptibility levels after infiltration-inoculation (Figure S2). Since Arabidopsis restricts bacterial invasion through stomatal closure [4], [7], we hypothesized that *lecrk-V.5* mutants increased resistance to surface-inoculation with bacteria is due to their ability to prevent bacterial entry inside leaves *via* stomata. To determine LecRK-V.5 possible function in stomatal immunity, we examined stomatal aperture of *lecrk-V.5* mutants upon *Pst* DC3000 inoculation. Stomata of buffer-treated epidermal peels of *lecrk-V.5* mutants were closed at levels similar to those observed in WT controls at 1 hpi with bacteria (Figure 2A and Figure S3A). In addition, the COR-dependent stomatal reopening that occurred in WT at 3 hpi was not observed in *lecrk-V.5* mutants (Figure 2A and Figure S3A). Complemented lines demonstrated a WT stomatal aperture in response to *Pst* DC3000 (Figure 2A), suggesting that mutations in *LecRK-V.5* caused the stomatal response phenotype observed in *lecrk-V.5* mutants. We also analyzed stomatal aperture in WT and lines overexpressing *LecRK-V.5* at 1, 1.5 and 3 hpi with *Pst* DC3000. Although stomatal closure in OE-1 and OE-2 was observed at 1 hpi with *Pst* DC3000, stomata reopened in overexpression lines at 1.5 hpi, a time point where WT stomata were still closed (Figure S3B). An early stomatal reopening may explain the increased susceptibility of OE-1 and OE-2 lines to bacteria.

WT Col-0 Arabidopsis are resistant to surface inoculation with COR-deficient mutants of *Pst* DC3000 (*Pst* DC3000 COR^−^), presumably because their stomata do not reopen upon infection [4], [22]. To further assess the possible role of LecRK-V.5 in stomatal immunity, lines overexpressing *LecRK-V.5* (OE-1 and OE-2) were dip-inoculated with *Pst* DC3000 COR^−^ bacteria and disease development was evaluated 3 days later. The defective virulence of *Pst* DC3000 COR^−^ observed in Col-0 was almost fully rescued in lines overexpressing *LecRK-V.5* (Figure 2B). We also analyzed stomatal aperture in WT and overexpression lines after *Pst* DC3000 COR^−^ inoculation. Lines overexpressing *LecRK-V.5* reopened stomata as early as 4 hpi (Figure 2C). Since Arabidopsis WT stomata do not reopen upon infection with *Pst* DC3000 COR^−^ (Figure 2C) [4], [22], stomatal reopening in OE-1 and OE-2 lines may explain their increased susceptibility to *Pst* DC3000 COR^−^ (Figure 2B). Taken together these data suggest a role for LecRK-V.5 in mediating stomatal movement in response to *Pst* DC3000 bacteria in Arabidopsis.

### A negative role for LecRK-V.5 in PAMP-induced stomatal closure

**Figure 3:**
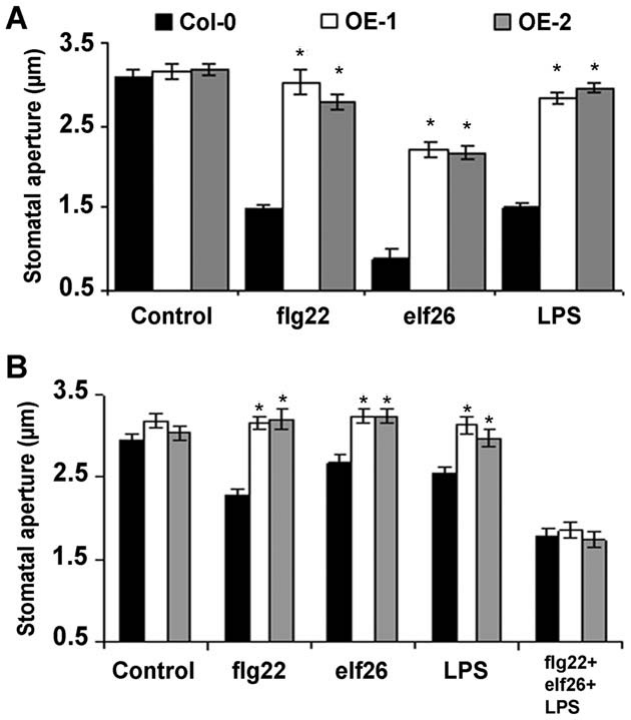
Altered PAMP-induced stomatal closure in lines overexpressing *LecRK-V.5*. (A) Stomatal apertures in epidermal peels of WT Col-0 and overexpression lines (OE-1 and OE-2) after 3 hrs of incubation with MES buffer (Control), 5 µM flg22, 5 µM elf26 or 100 ng.µL^−1^ LPS. (B) Stomatal apertures were measured on epidermal peels incubated in MES buffer (Control), 1 µM flg22, 1 µM elf26, 10 ng.µL^−1^ LPS or 1 µM flg22, 1 µM elf26 and 10 ng.µL^−1^ LPS together. Results are shown as mean of ≥60 stomata measurements ± SE. Asterisks indicate significant differences between WT and OE lines based on a *t* test (*P*<0.001). All experiments were repeated at least three times with similar results. hpi, hour post inoculation.

To further define the role of LecRK-V.5 during stomatal immunity, we tested the ability of lines overexpressing *LecRK-V.5* to respond to PAMP-mediated stomatal closure. Epidermal peels were incubated with the PAMPs flg22, the active elongation factor Tu epitope elf26 and lipopolysaccharides (LPS), which promote stomatal closure [4], [9]. Stomatal closure was strongly reduced in OE-1 and OE-2 lines after PAMP treatments at concentrations that are usually used to induce stomatal closure in WT controls (Figure 3A) [4]. Treatments with higher concentrations of flg22 or elf26 partially restored OE-1 and OE-2 sensitivity to these PAMPs (Figure S4A, B). By contrast, these transgenics demonstrated a defective stomatal response to LPS concentrations up to 200 ng.µL^−1^ (Figure S4C). Surprisingly, stomata of lines overexpressing *LecRK-V.5* responded similarly to WT control to low concentrations of these PAMPs when all 3 PAMPs were applied together (Figure 3B). This observation may explain the observed stomatal closure of OE-1 and OE-2 after *Pst* DC3000 inoculation (Figure S3B).

### *lecrk-V.5* mutants demonstrate normal apoplastic PTI responses

**Figure 4:**
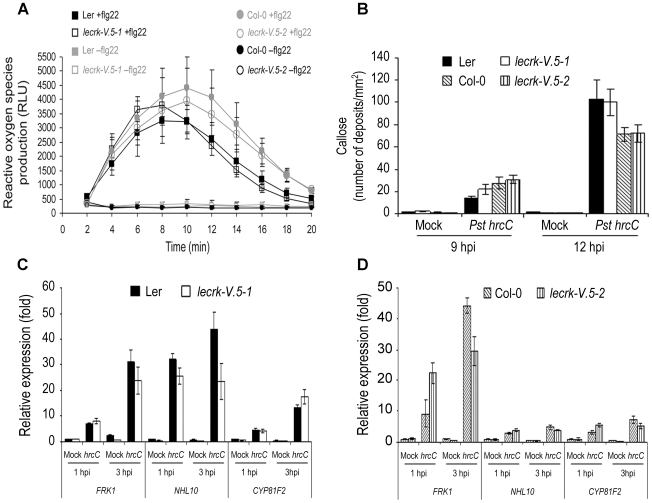
Apoplastic PTI responses in *lecrk-V.5* mutants. (A) Production of reactive oxygen species in Arabidopsis leaves after treatment with 1 µM flg22 as relative light units (RLU). Values represent averages ± SE (n = 6). (B) Callose deposition in WT or *lecrk-V.5* mutants leaves infiltrated with 10 mM MgSO4 (Mock) or *Pst* DC3000 *hrcC* (*Pst hrcC*). Data represent the number of callose deposits per square millimeter ± SD. Differences were not significantly different to WT based on a *t* test (*P*<0.01). (C, D) *FRK1*, *NHL10*, *CYP81F2* expression levels in WT or *lecrk-V.5* mutant seedlings soaked in 10 mM MgSO_4_ (Mock) or 1×10^7^ cfu.ml^−1^ *Pst* DC3000 *hrcC* (*hrcC*). Transcript levels were determined by qRT-PCR and normalized to both *EF-1* and *UBQ10*. Bars indicate SD (n = 9). All experiments were repeated 3 times with similar results.

Stomatal closure is only one aspect of the PTI response. To determine whether apoplastic PTI responses were constitutively elevated in *lecrk-V.5* mutants, we analyzed the production of H_2_O_2_ as an early response to PAMPs in *lecrk-V.5-1* and *lecrk-V.5-2* leaves in response to flg22 [23]. ROS production after flg22 treatment was at a WT level in both *lecrk-V.5* mutants (Figure 4A). We also examined callose deposition [24] and accumulation of the PTI-responsive *FRK1*, *NHL10* and *CYP81F2* [25] after inoculation with the Type III secretion system deficient bacterial mutant *Pst* DC3000 *hrcC* (CB200) [26]. Callose deposition and PTI-gene expression levels were not constitutively elevated in *lecrk-V.5* mutants and inoculation with *Pst* DC3000 *hrcC* induced WT callose deposition (Figure 4B) and PTI-gene expression levels (Figure 4C, D). These data suggest that enhanced resistance of *lecrk-V.5* mutants to *Pst* DC3000 is not correlated with a constitutively activated apoplastic PTI or boosted ROS production, increased callose deposition or PTI-responsive gene expression levels upon PTI activation in the leaves.

### LecRK-V.5 is localized at the plasma membrane and expressed in stomatal guard cells

**Figure 5:**
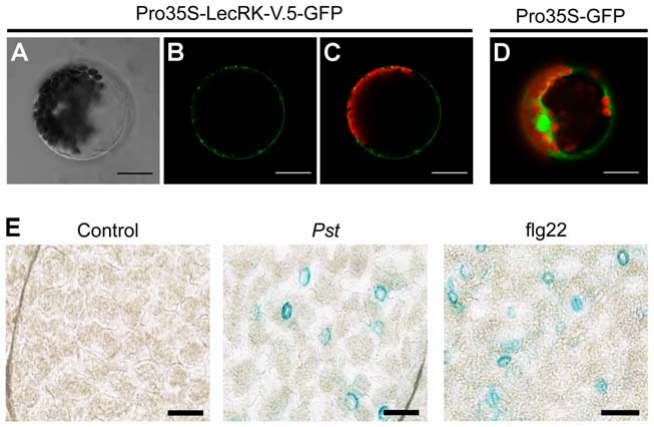
LecRK-V.5 is localized at the plasma membrane and is expressed at guard cells upon PTI activation. (A, B and C) Subcellular localization of LecRK-V.5-GFP fusion protein in Arabidopsis mesophyll protoplasts. *LecRK-V.5-GFP* expression was driven by the cauliflower mosaic virus 35S promoter and transiently expressed in Arabidopsis mesophyll protoplasts. The bright-field image (A), images of the GFP fluorescence (green) only (B), and the overlap image of the GFP (green) and chlorophyll (red) fluorescence (C) are presented. (D) Photo of a control protoplast expressing GFP alone. Bars = 20 µm. (E) The activity of *LecRK-V.5* promoter was detected by GUS staining in guard cell 15 min after inoculation with 1×10^8^ cfu.ml^−1^ *Pst* DC3000 (*Pst*) or 1 µM flg22 treatment. Bar represents 20 µm.

To determine the subcellular localization of LecRK-V.5, the LecRK-V.5-GFP fusion protein driven by the cauliflower mosaic virus 35S promoter was transiently expressed in Arabidopsis mesophyll protoplasts. Confocal imaging indicates that the fluorescence signal is confined to a ring external to the chloroplast signal, while the control protoplasts expressing GFP alone showed a nuclear and cytoplasmic GFP localization (Figure 5A–D). Since LecRK-V.5 was previously detected in plasma membrane fraction [17], our data confirm that LecRK-V.5 is localized at the plasma membrane.

We then asked whether LecRK-V.5 expression is localized at stomatal guard cells upon stomatal immunity activation. *LecRK-V.5* promoter GUS analyses indicated that *LecRK-V.5* is induced specifically in stomatal guard cells 15 min after *Pst* DC3000 inoculation or flg22 treatment (Figure 5E). This expression pattern suggests a role for LecRK-V.5 in stomatal movement upon pathogen infection.

### LecRK-V.5 regulates stomatal closure upstream of COR site of action

**Figure 6:**
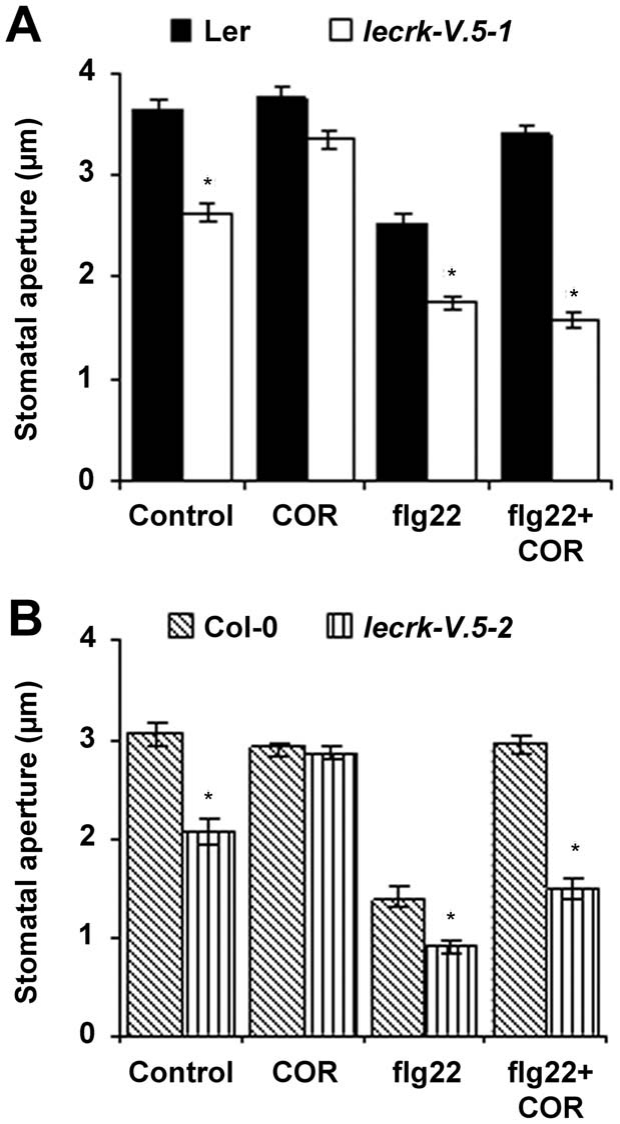
Effects of COR on flg22-mediated stomatal closure in *lecrk-V.5* mutants. (A, B) Stomatal aperture in epidermal peels of WT (Ler and Col-0) and *lecrk-V.5* mutants exposed to MES buffer (Control), 0.5 ng.µL^−1^ COR, 5 µM flg22 or 5 µM flg22 and 0.5 ng.µL^−1^ COR together (flg22+COR) for 3 hrs. Results are shown as mean of ≥60 stomata measurements ± SE. Asterisks indicate significant differences between WT and mutants based on a *t* test (*P*<0.001). All experiments were repeated at least three times with similar results.

COR counteracts PAMP-induced stomatal closure and reverses flg22 inhibition of inward K^+^ channels downstream of ROS production [4], [6]. To determine whether LecRK-V.5 regulates stomatal closure upstream or downstream of COR site of action, we evaluated stomatal apertures in epidermal peels of *lecrk-V.5* mutants treated with COR. Treatments with COR only opened closed stomata in *lecrk-V.5-1* and *lecrk-V.5-2* (Figure 6A, B), suggesting that LecRK-V.5 negatively regulates PAMP-induced stomatal closure upstream of COR site of action. Although *Pst* DC3000 bacteria produce COR [4], inoculation with *Pst* DC3000 did not open stomata of *lecrk-V.5* mutants (Figure 2A). Epidermal peels were thus treated with flg22 alone or flg22 together with COR. The PAMP flg22 induced stomatal closure in WT plants and further closure in both *lecrk-V.5* mutants (Figure 6A, B). However, COR did not counteract flg22-mediated stomatal closure in *lecrk-V.5* mutants while stomata of flg22-treated WT controls did reopen after COR treatment (Figure 6A, B). Together, these data suggest that mutations in *LecRK-V.5* inhibit the COR-dependent reopening of stomata during PAMP-induced stomatal closure.

### Both *lecrk-V.5* mutants accumulate high levels of ROS in guard cells

**Figure 7:**
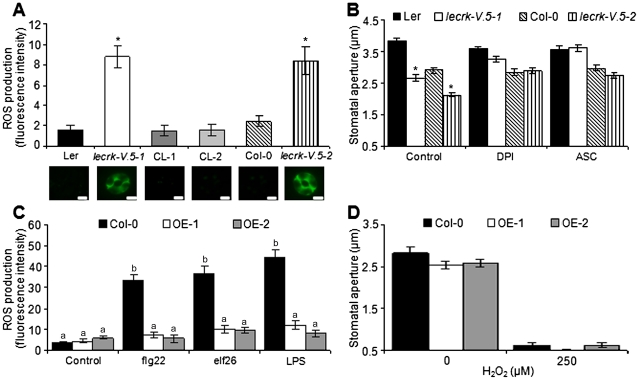
Role of LecRK-V.5 in ROS-mediated stomatal closure. (A) ROS detected by H_2_DCFDA fluorescence in non-treated WT (Ler and Col-0), *lecrk-V.5* mutants and two complemented line (CL-1 and CL-2) guard cells. A representative stoma is shown. Bars represent 7.5 µm. (B) Stomatal aperture in epidermal peels of WT (Ler and Col-0) and *lecrk-V.5* mutants exposed to MES buffer (Control), 20 µM DPI or 1 mM ASC for 3 hrs. (C) ROS detected by H_2_DCFDA fluorescence in guard cells of WT Col-0 and lines overexpressing *LecRK-V.5* (OE-1 and OE-2) after treatments with MES buffer (Control), 5 µM flg22, 5 µM elf26 or 100 ng.µL^−1^ LPS. (D) Stomatal aperture in epidermal peels of WT Col-0 and overexpression line OE-1 and OE-2 after 3 h incubation with H_2_O_2_. For all experiments, results are shown as mean ± SE. In (A), (B) and (D) asterisks indicate a significant difference to WT control based on a *t* test analysis (n≥60; *P*<0.001). In (C), different letters indicate statistically significant differences compared with the non-treated WT Col-0 (Fisher's Least Significant Difference test; n≥60; P<0.05). All experiments were repeated at least three times with similar results.

To further clarify the role of LecRK-V.5 in the stomatal response, ROS levels in *lecrk-V.5-1* and *lecrk-V.5-2* guard cells were analyzed with the fluorescent dye 2′,7′-dichlorofluorescein diacetate (H_2_DCFDA) [27], [28]. Microscopy and fluorescence emission analyses revealed a higher level of ROS in guard cells of both *lecrk-V.5* mutants (Figure 7A). WT controls and complemented lines demonstrated similar levels of ROS production (Figure 7A), suggesting that mutations in *LecRK-V.5* caused ROS accumulation observed in *lecrk-V.5* mutants. Diphenylene iodium chloride (DPI), an inhibitor of NADPH oxidases known to inhibit ABA-induced stomatal closure [28], was used to test a possible role for NADPH oxidases in *lecrk-V.5* stomatal phenotype. DPI treatments induced stomatal opening in *lecrk-V.5-1* and *lecrk-V.5-2* (Figure 7B). To further evaluate the role of ROS, plants were treated with ascorbic acid (ASC), a chemical known to reduce ROS levels [27]. Treatment with ASC also opened constitutively closed stomata in *lecrk-V.5* mutants (Figure 7B). These results suggest that over-accumulation of ROS is responsible for the constitutive stomatal closure observed in *lecrk-V.5* mutants. Treatments with PAMPs such as flg22, elf26 or LPS boosted guard cell ROS production at WT levels in *lecrk-V.5-1* (Figure S5). By contrast, treatments with these PAMPs increased guard cell ROS levels in WT, but no increase of ROS production was observed in Arabidopsis overexpressing *LecRK-V.5* (Figure 7C). Furthermore, H_2_O_2_-induced stomatal closure was normal in overexpression lines (Figure 7D) suggesting that LecRK-V.5 does not influence stomatal closure signaling downstream of ROS biosynthesis. LecRK-V.5 may thus function upstream of ROS production in guard cell movement.

### LecRK-V.5 role in stomatal immunity is mechanistically linked to ABA signaling

**Figure 8:**
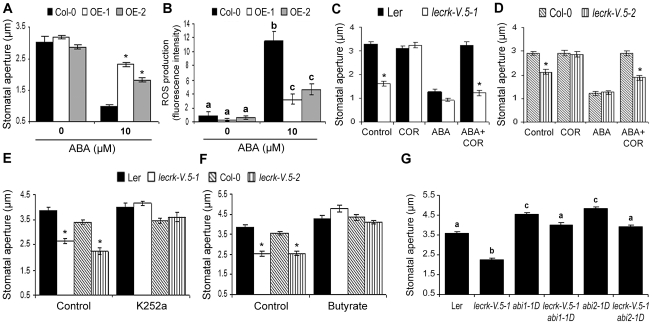
LecRK-V.5 role in stomatal immunity is mechanistically linked to ABA signaling. (A) Stomatal apertures in epidermal peels of WT Col-0 or *LecRK-V.5* overexpression transgenics OE-1 and OE-2 were measured after 3 hrs incubation in ABA. (B) H_2_DCFDA-detected ROS production after ABA treatments. (C, D) Stomatal apertures in epidermal peels of WT (Ler and Col-0) and *lecrk-V.5* mutants exposed to MES buffer (Control), 0.5 ng.µL^−1^ COR, 10 µM ABA, or 10 µM ABA together with 0.5 ng.µL^−1^ COR (ABA+COR) for 3 hrs. (E, F) Arabidopsis WT (Ler and Col-0) or *lecrk-V.5* mutants epidermal peels were floated in MES buffer (Control) or 1 µM K252a (E) or 0.5 mM butyrate (F) for 3 hrs before stomatal aperture measurement. (G) Stomatal aperture in *lecrk-V.5-1 abi1-1D* and *lecrk-V.5-1 abi2-1D* double mutants. For B and G, different letters indicate statistically significant differences compared with WT (Fisher's Least Significant Difference test; n≥60; P<0.05). For A, C, D, E and F, results are shown as mean ± SE (n≥60) and asterisks indicates significant differences between WT and mutant/OE based on a *t* test (*P*<0.001). All experiments were repeated at least three times with similar results.

Innate immunity-mediated stomatal closure depends on ABA signaling [4], [6], [22]. To evaluate whether LecRK-V.5 plays a role in the ABA-mediated stomatal closure, stomatal apertures after treatment with ABA were assessed in lines overexpressing *LecRK-V.5*. Stomata of such lines were greatly compromised in their ability to respond to ABA (Figure 8A). Furthermore, overexpression lines demonstrated reduced ROS production after ABA treatment (Figure 8B). COR inhibits ABA-induced stomatal closure [4], [6]. We thus evaluated the possibility that *lecrk-V.5* mutants were resistant to COR-inhibition of stomatal closure upon ABA treatments. Similarly to PAMP-induced stomatal closure (Figure 6A, B), COR treatments did not open ABA-treated *lecrk-V.5-1* and *lecrk-V.5-2* stomata while reopening in WT controls was observed (Figure 8C, D). To further evaluate the role of LecRK-V.5 in ABA-mediated stomatal closure, we manipulated the ABA-mediated regulation of guard cell pH. Butyrate which causes an acidification of cytoplasm, and K252a, a protein kinase inhibitor, are able to suppress ABA-induced alkalization occurring upstream of ROS production in guard cells [29]. Both compounds opened constitutively closed *lecrk-V.5-1* and *lecrk-V.5-2* stomata (Figure 8E, F). Double mutants generated by crossing *lecrk-V.5-1* with ABA-insensitive mutants *abi1-1D* and *abi2-1D* [30], [31] demonstrated a WT stomatal aperture further indicating that LecRK-V.5 functions in ABA signaling (Figure 8G). In addition to ABA, the plant hormones jasmonate (JA) and SA play a positive role in stomatal closure [7], [29], [32]. Both OE-1 and OE-2 lines demonstrated WT levels of stomatal closure in response to these two phytohormones (Figure S6). Collectively, these results suggest that LecRK-V.5 interferes with ABA- but not with JA- or SA-mediated stomatal closure signaling.

## Discussion

As a part of the plant innate immune system, stomata play an active role in limiting bacterial entry into plant tissues and subsequent disease symptoms [4], [5]. A rapid stomatal closure occurs upon bacterial challenge and some pathogens have evolved virulence factors such as COR for *Pst* DC3000 to overcome plant stomatal immunity [4], [33], [34]. Plants lacking a functional *LecRK-V.5* displayed enhanced resistance to *Pst* DC3000 surface-inoculation, but were susceptible as WT after bacteria-infiltration, an inoculation method that bypasses the epidermal barrier. These results suggest that disruption of *LecRK-V.5* affects early Arabidopsis defenses by restricting bacterial entry into leaves. Differences between surface- and infiltration-inoculation were also observed in Arabidopsis mutants defective in bacteria-induced stomatal closure [4], [22], [35], [36]. Since *lecrk-V.5* mutants demonstrated WT apoplastic PTI (Figure 4), closed stomata and inhibition of COR-dependent stomatal reopening is the most straightforward explanation for the enhanced resistance phenotype of *lecrk-V.5* mutants to *Pst* DC3000 surface inoculation. *LecRK-V.5* overexpression lines were more susceptible than WT plants to bacteria, notably to *Pst* DC3000 COR^−^ dip-inoculation (Figure 2B). Enhanced susceptibility to WT *Pst* DC3000 was correlated with an earlier reopening of stomata, further pointing for a role of LecRK-V.5 in stomatal immunity. The reopening of stomata after inoculation with *Pst* DC3000 COR^−^ suggests the existence of a plant stomatal reopening mechanism positively modulated by LecRK-V.5. Taken together these data suggest that LecRK-V.5 negatively regulates Arabidopsis resistance to bacteria through fine-tuning of stomatal immunity.

Treatments with PAMPs such as flg22, elf26, or LPS induce rapid stomatal closure and the *fls2* mutant is defective in flg22-induced stomatal closure implying the PTI response in stomatal immunity [4], [9]. Similarly to the *fls2* mutant, all mutants defective in PAMP-induced stomatal closure described so far are highly susceptible to surface-inoculation with *Pst* DC3000 or *Pst* DC3000 COR-deficient mutant bacteria most likely because of a defect in bacteria-induced stomatal closure [4], [22], [35], [36]. Lines overexpressing *LecRK-V.5* were defective in PAMP-induced stomatal closure, suggesting a negative role for LecRK-V.5 during stomatal immunity. Treatments of over-expression lines with low concentrations of elf26, LPS and flg22 that did not induce stomatal closure when applied individually, triggered stomatal closure when applied all together (Figure 3B). This observation suggests that different PAMPs additively activate the stomatal immunity response modulated by LecRK-V.5. It likely explains why over-expression lines exhibit a WT bacterium-induced stomatal closure. Other aspects of the Arabidopsis PTI response such as the flg22-triggered oxidative burst, *Pst* DC3000 *hrcC*-mediated callose deposition and up-regulation of PTI marker genes were not affected in *lecrk-V.5* mutants. These apoplastic PTI responses are mostly mediated by mesophyll cells [1], [22], [36]. The recently isolated *scord5* mutant also shows a defective stomatal immunity but exhibits WT apoplastic immunity [36]. Our data therefore confirm recent findings indicating that stomatal immunity can be distinguished from the general PTI response [36]. Localized expression of *LecRK-V.5* upon PTI activation at stomatal guard cells may explain the specific role of LecRK-V.5 in stomatal immunity.

The signaling pathways leading to bacteria and PAMP-induced stomatal closure downstream of PRRs (e.g. FLS2) remains unclear. Analyses of SA-deficient *nahG* transgenics, SA-biosynthetic mutant *sid2*/*eds16* and ABA-deficient mutant *aba3* indicate that SA and ABA biosynthesis are required for PAMP-induced stomatal closure [4]. The ABA signaling components OST1, ABI1, GPA1 and OST2 are also required for bacteria- and PAMP-induced stomatal closure [4], [6], [34], [5], [36]. These studies illustrate the complexity of hormonal crosstalks involved in stomatal immunity. *LecRK-V.5* overexpression lines were defective in ABA-mediated stomatal closure but not in SA- and JA-mediated stomatal closure. ABA-induced ROS production was also affected in lines over-expressing *LecRK-V.5*. As it was proposed for ABI1, OST1 and GPA1 [4], [6], [34], [35], [36], LecRK-V.5 appears to specifically function in guard cell ABA signaling pathway downstream of PAMP perception. LecRK-V.5 may thus act at a specific branch involving ABA for the control of stomatal immunity.

During stomatal closure, ABA induces ROS accumulation, which activates plasma membrane calcium channels, induces increase in cytosolic Ca^2+^, and triggers stomatal closure [28], [37], [38]. In this study, constitutive high levels of ROS in guard cells were correlated with constitutively closed stomata in *lecrk-V.5* mutants. This phenotype was reverted by treatments with an inhibitor of NADPH oxidase (DPI) [28] or ASC, a chemical that reduces ROS levels [27]. Increase in ROS levels and stomatal closure after PAMP and ABA treatments were impaired in lines overexpressing *LecRK-V.5*. In addition, suppression of ABA-induced alkalization that takes place upstream of ROS production [29] opened closed stomata of *lecrk-V.5* mutants. Recent studies suggest that constitutive ROS accumulation does not induce stomatal closure, but ROS accumulation mediated by ABA does [39], [40]. Collectively these observations suggest a role for LecRK-V.5 upstream of ROS production in the ABA-mediated stomatal closure signaling. Importantly, H_2_O_2_-induced stomatal closure in *LecRK-V.5* overexpression lines was not affected. Thus LecRK-V.5 probably does not disrupt the pathway downstream of ROS biosynthesis. Since generation of H_2_O_2_ in guard cells and stomatal closure in response to ABA occurs *via* NADPH oxidases Rboh [10], LecRK-V.5 may act upstream of Atrboh in PAMP- and bacteria-mediated ROS production. By preventing guard cell ROS accumulation, LecRK-V.5 may function in ROS homeostasis to regulate H_2_O_2_ content in guard cells. Plants likely have evolved a mechanism to reverse PAMP-, bacteria- or ABA-triggered stomatal closure to prevent long-term detrimental effects on CO_2_ uptake and photosynthesis. LecRK-V.5 may be one component of this protective mechanism.

**Figure 9:**
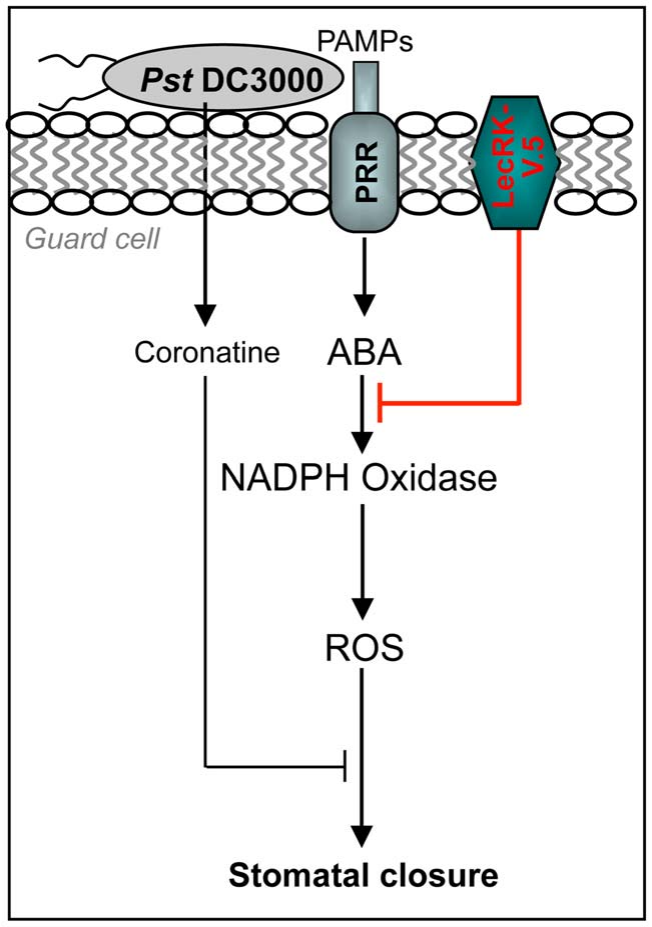
Model of LecRK-V.5 role in PAMP-induced stomatal closure. This model is based on the information provided in this study and references cited in the Discussion. PAMPs are perceived by pattern recognition receptors (PRRs) in the guard cell. PAMPs perception is mechanistically linked to ABA-regulated stomatal closure *via* ROS production by NADPH oxidase. The virulence factor COR is secreted by *Pst* DC3000 to interfere with stomatal closure by reverting flg22-inhibition. LecRK-V.5 negatively regulates PAMP-mediated stomatal closure downstream of ABA but upstream of ROS production.

In this study, the L-type lectin receptor kinase LecRK-V.5 was identified as a key player in stomatal immunity. We propose a model in which LecRK-V.5 negatively regulates the signaling pathway leading to PTI-mediated stomatal closure downstream of ABA and upstream of NADPH oxidase to fine tune ROS accumulation in stomatal guard cells (Figure 9). The OST1/SnRK2E/SnRK2.6 protein kinase involved in PAMP-induced stomatal closure [4] was initially identified as a positive regulator upstream of ROS in guard cell ABA signaling [41]. A recent study showed that OST1 interacts with and phosphorylates AtrbohF [42]. Further studies are required to identify protein partners of LecRK-V.5 and implicated in PTI-mediated stomatal closure signaling upstream of ROS production.

## Materials and Methods

### Biological materials and growth conditions

*Arabidopsis thaliana* (L. Heyhn.) ecotypes Columbia (Col-0) and Landsberg *erecta* (Ler), and derived mutant lines were grown as previously described [43]. The *lecrk-V.5-1* (GT12539) Ds transposon insertion line (Ler) is from the Cold Spring Harbor Laboratory (http://genetrap.cshl.org/) and the *lecrk-V.5-2* (GK-623G01) T-DNA insertion line (Col-0), *abi1-1D* and *abi2-1D* were obtained from the Nottingham Arabidopsis Stock Centre (NASC, http://arabidopsis.info/). Details about PCR analyses performed to screen for homozygous mutants are described under “Gene Expression Studies”. Bacterial strains *Pst* DC3000, *Pst* DC3000 COR^−^ (DB29) and *Pst* DC3000 *hrcC* mutant (CB200) were provided by B.N. Kunkel (Washington University, St. Louis, USA) [26]. *Pst* bacteria were cultivated at 28°C, 340 rpm in King's B medium containing rifampicin (*Pst* DC3000), rifampicin, spectinomycin and kanamycin (DB29) or rifampicin and kanamycin (CB200).

### Bacterial infection assays

Bacterial disease assays were conducted as previously described [44]. For surface inoculation, plants were dipped in a bacterial solution of 1×10^7^ cfu.ml^−1^ containing 0.01% Silwet L-77 (Bioman Scientific Co., Ltd.). Alternatively, three fully expanded leaves per plant were infiltrated on the abaxial surface with *Pst* DC3000 at a concentration of 1×10^5^ cfu.ml^−1^ using a needleless 1 ml syringe.

### Plasmid constructions and generation of transgenic plants

All cloning experiments were performed using the genomic clone AF001168 kindly provided by Christine Hervé (INRA Toulouse, France). According to [18], 707 pb of the *LecRK-V.5* promoter was PCR amplified using the GW-pRK-F (5′-CTGCAACAATTGGGAGGAGGG-3′) and GW-pRK-R (5′-GTTCACGAGACTTTGGTGGGTG-3′) primers. The coding sequence (CDS) of *LecRK-V.5* was PCR amplified using the GW-RK-F (5′-ATGTCTCGTGAACTTATTATTCTCTGCC-3′) and GW-RK-R (5′-TCAGCGGCCGTGGGAGACAA-3′) primers. The resulting PCR products were directly cloned by the TOPO cloning reaction into the pCR8/GW/TOPO vector (Invitrogen) following manufacturer's instructions. The promoter and CDS (without stop codon) of *LecRK-V.5* was PCR amplified with the GW-attB1-RK-F (5′-GGGGACAAGTTTGTACAAAAAAGCAGGCTCTGCAACAATTGGGAGGAGGG-3′) and GW-attB2-RK-R (5′-GGGGACCACTTTGTACAAGAAAGCTGGGTCGCGGCCGTGGGAGACAAAAG-3′) primers and recombined by the BP reaction into the pDNOR221 vector (Invitrogen). The *LecRK-V.5* promoter was sub-cloned by LR reaction in the pMDC163 vector [45] to produce the *ProLecRK-V.5::GUS* construct. The *LecRK-V.5* CDS and promoter plus CDS were then introduced into the gateway pEarleyGATE100, pEarleyGATE201, pEarleyGATE301 and pMDC83 binary vectors [46] to respectively obtain the *35S::LecRK-V.5*, *35S::HA-LecRK-V.5*, *ProLecRK-V.5::LecRK-V.5-HA* and *35S::LecRK-V.5-GFP* constructs. The fidelity of all constructs was confirmed by sequencing. Arabidopsis plants were transformed using the GV3101 strain of *Agrobacterium tumefaciens* according to the floral dip protocol [47]. Transgenic insertion lines with single insertion loci were selected on 1/2 MS plates containing 50 µM Glufosinate ammonium (Fluka) and raised to homozygous T3 lines.

### Generation of double mutants

The *lecrk-V.5-1 abi1-1D* and *lecrk-V.5-1 abi2-1D* double mutants were selected in the F_2_ progeny of crosses between the two corresponding homozygous parents. Verifications of genotypes involving *abi1-1* and *abi2-1* crosses were performed as described [31].

### Gene expression studies

Semi-quantitative PCR and qRT-PCR were as described [43], with some modifications. Total RNA was extracted and purified using Qiagen RNeasy plant Mini Kit with additional genomic DNA cleanup using Qiagen RNase-Free DNase Set. For cDNA synthesis, RNA was first diluted to 2 µg in a total volume of 22 µL DEPC water and denatured at 65°C for 5 min. Eighteen point five µL of master mix (1× M-MLV buffer, 1 mM dNTP, 5 µM OligoT, 100 U M-MLV reverse transcriptase, [Invitrogen]) was added into each tube and then incubated at 37°C for 1 hr, 70°C for 10 min. cDNA was diluted 5-fold before real-time PCR or semi-quantitative PCR. PCR amplification was done with 2 µL of the first-strand cDNA as template, 1 unit of Taq DNA polymerase (Viogene), 156 µM dNTP and 0.5 µM of primers in a total volume of 20 µL. Primers used for screening of homozygous mutants were GT12539-F (5′-TCGGCTTCAACGTTTACTTC-3′) and GT12539-R (5′-CGATGGAAAGCCTCATTACC-3′) for the *lecrk-V.5-1* mutant (P1) and Salk_083045-F (5′-ATGGGTTGGTTAGTTAATGG-3′) and Salk_083045-R (5′-CCTCGCATTCATTTCATTGTC-3′) for the *lecrk-V.5-2* mutant (P2). The cycling conditions were 94°C for 5 min for one initial step followed by 94°C for 30 s, 60°C for 30 s and 72°C for 1 min, for 40 cycles. The PCR was terminated with one extra step at 72°C for 10 min. iQ SYBR Green supermix [Bio-RAD] (2 µL of cDNA, 9 µL SYBR Green supermix, 5 µL filtered water, 1 µL of 10 µM forward primer, 1 µL of 10 µM reverse primer, in a total volume of 18 µL per well) was employed for real-time PCR analysis. The cycling conditions were composed of an initial 3 min denaturation step at 95°C, followed by 40 cycles of 95°C for 30 s, 54°C for 35 s, 72°C for 35 s (iQ5 Real-Time PCR Detection System, Bio-RAD). Melting curve was run from 55°C to 95°C with 10-second time interval to ensure the specificity of product. Data were analyzed using Bio-Rad iQ5 software (version 2.0). Elongation factor 1 (*EF-1*) and ubiquitin (*UBQ10*) were used as reference genes for normalization of gene expression levels in all samples. The WT without any treatment or mock treatment were considered as controls (expression level = 1) in each experiment. qRT-PCR forward and reverse primers of each gene were as follows: 5′-GAATGGAGTTTCACAGCTACCA-3′ and 5′-GTCGGTTAACTCCAATGAACTC-3′ for *LecRK-V.5* expression in complemented lines (CL-1 and CL-2); 5′-TCATGGCATACTTCGTCTCAC-3′ and 5′-CTCATCGACAGGTGTCATCT-3′ for *LecRK-V.5* expression in overexpression lines (OE-1 and OE-2); 5′-AAA TGG AGA GAG CAA CAC AAT G-3′ and 5′-ATC GCC CAT TCC AAT GTT AC-3′ for *CYP81F2* (At5g57220); 5′-TTC CTG TCC GTA ACC CAA AC-3′ and 5′-CCC TCG TAG TAG GCA TGA GC-3′ for *NHL10* (At2g35980); 5′-GCC AAC GGA GAC ATT AGA G-3′ and 5′-CCA TAA CGA CCT GAC TCA TC-3′ for *FRK1* (At2g19190); 5′-TGA GCA CGC TCT TCT TGC TTT CA-3′ and 5′-GGT GGT GGC ATC CAT CTT GTT ACA-3′ for *EF-1* (At1g07920) and 5′-GGCCTTGTATAATCCCTGATGAAT-3′ and 5′-AAAGAGATAACAGGAACGGAAACA-3′ for *UBQ10* (At4g05320).

### Subcellular localization in protoplast

For transient expression of the GFP fusion proteins, constructs expressing *35S::LecRK-V.5-GFP* and vector alone were co-transfected into Arabidopsis mesophyll protoplasts according to a previously described protocol [48]. Briefly, leaves from 5-week-old plants were digested in an enzyme solution containing 1.5% cellulose R10 (Yakult Pharmaceutical Ind. Co.) and 0.3% macerozyme R10 (Yakult Pharmaceutical Ind. Co.). Transfected protoplasts were incubated overnight under light at room temperature. Confocal laser scanning microscopy with excitation at 488 nm and emission at 500–530 nm (Leica TCS SP5 Confocal, Leica, Wetzlar, Germany) was carried out to visualize subcellular localization of LecRK-V.5-GFP. Autofluorescence was monitored at 488 nm, and transmission images were collected in parallel.

### Stomatal experiments

Leaf peels were collected from the abaxial side of fully expanded leaves and floated in stomatal buffer (10 mM MES-KOH, 30 mM KCl, pH 6.15) for 2.5 h under light (100 µmol m^−2^ s^−1^) to ensure that most of stomata were opened before treatments [4]. Purified chemical lipopolysaccharide (LPS from *P. aeruginosa*, Sigma), flg22 peptide (Biomer Technology, CA), elf26 (Biomer Technology, CA) or COR (Sigma) were used at indicated concentrations. flg22 and elf26 were diluted in 10 mM MgSO_4_. COR and LPS were respectively diluted in milliQ water and in MES buffer containing 0.25 mM MgCl_2_ and 0.1 mM CaCl_2_ [5]. ABA, methyl jasmonate (MeJA) and SA used at indicated concentrations were dissolved in 10% ethanol (Sigma). Diphenyleneiodonium chloride (DPI, Sigma) and K252a (Sigma) were dissolved in dimethylsulfoxide (DMSO). Ascorbic acid (ASC) and sodium butyrate (Butyrate, Sigma) were prepared in milliQ water. Mock controls were MES buffer containing 0.1% ethanol for MeJA, ABA and SA, 0.1% DMSO for DPI and K252a, and milliQ water for ASC and Butyrate. Bacterial concentration used was 1×10^8^ cfu.ml^−1^ in 10 mM MgSO_4_. Stomatal apertures were measured as described [43].

### Monitoring ROS in guard cells

2′,7′-dichlorodihydrofluorescein diacetate (H_2_DCFDA) fluorescence analysis were performed essentially as described [49]. After 2.5 hrs incubation in stomatal buffer, epidermal peels were transferred to 50 µM H_2_DCFDA in 10 mM Tris-HCl pH 7.2 for 15 min. Excess H_2_DCFDA was then removed by washing 3 times in 10 mM Tris-HCl pH 7.2. Then, PAMPs (5 µM flg22, 5 µM elf26 or 100 ng.µL^−1^ LPS) or 10 µM ABA were added to the incubation buffer for 10 min. H_2_DCFDA fluorescence was observed with an Olympus BX51 fluorescence microscope with an excitation at 460–480 nm and emission at 495–540 nm. Images were captured with an Olympus DP72 digital camera linked to the Olympus DP2-BSW software. Fluorescence was analyzed using ImageJ 1.42 sofware.

### Apoplastic oxidative burst evaluation

ROS released by leaf tissue was assayed as described [23]. Leaf of 5-week-old Arabidopsis plants were cut in 2 mm^2^ pieces and floated overnight in water. ROS production was triggered with 1 µM flg22 applied together with 100 µM luminol (Sigma) and 1 µg/mL of horseradish peroxidase (Sigma). Luminescence was measured by a Centro LB 962 microplate luminometer (Berthold Technologies) for 20 min after addition of flg22.

### Gus staining

Surface-sterilized seeds were sown on 1/2 MS agar plates and cold-treated at 4°C in the dark for 3 days. The plates were then moved to germination conditions 22–24°C day, 17–19°C night temperature under a 15-h-light/9-h-dark photoperiod. Ten-day-old seedlings were transferred to 12 well plates containing liquid 1/2 MS. After overnight incubation, seedlings were treated with 1×10^8^ cfu.ml^−1^ *Pst* DC3000 or with 1 µM flg22 for 15 min. β-glucuronidase activity was determined as described [50].

### Callose staining

Arabidopsis were syringe infiltrated with 1×10^8^ cfu.ml^−1^ *Pst* DC3000 *hrcC* or 10 mM MgSO_4_ as a control. Nine leaf discs from 3 different plants were selected for analyses. Harvested leaf samples were cleared overnight by incubation in 95% ethanol at room temperature and then washed three times (2 hrs for each washing) with sterilized water. Cleared leaves were stained with 0.01% aniline blue in 0.15 M phosphate buffer (pH 9.5) for 24 hrs. Callose deposits were visualized under ultraviolet illumination using an Olympus DP72 digital camera linked to the Olympus DP2-BSW software. Callose deposits were counted using the “analyze particles” function of ImageJ 1.42 sofware.

### Growth conditions for PTI marker gene expression study

Surface-sterilized seeds were sown on 1/2 MS agar plates and cold-treated at 4°C in the dark for 3 days. The plates were then moved to germination conditions 22–24°C day, 17–19°C night temperature under a 15-h-light/9-h-dark photoperiod. Ten-day-old seedlings were transferred to 24 well plates. Three seedlings per well were soaked in 200 µL liquid 1/2 MS containing 1% sucrose. After incubation overnight, seedlings were treated with 1×10^8^ cfu.ml^−1^ *Pst* DC3000 *hrcC* mutant (CB200) for 1 hr and 3 hrs.

### Accession numbers

Sequence data from this article can be found in the Arabidopsis Genome Initiative under accession number(s): *LecRK-V.5* (At3g59700), *FRK1* (At2g19190), *NHL10* (At2g35980), *CYP81F2* (At5g57220), *EF-1* (At1g07920), *UBQ10* (At4g05320), *ABI1* (At4g26080) and *ABI2* (At5g57050).

## Supporting Information

Figure S1 ***LecRK-V.5*** **expression levels in transgenic lines.** Relative expression levels in WT (Ler) and two complemented lines (CL-1 and CL-2) and WT (Col-0) and two overexpression lines (OE-1 and OE-2). Transcript levels were determined by qRT-PCR and normalized to both *EF-1* and *UBQ10*. Expression levels were compared to WT controls with a defined expression value of 1. Bars indicate SD (n = 6). Experiments were repeated 3 times with similar results.
(TIF)

Figure S2 **Susceptibility of** ***lecrk-V.5*** **mutants to** ***Pst*** **DC3000 infiltration-inoculation.** Bacterial growth (colony forming units (cfu) per cm leaf area) was determined in Ler, Col-0 and *lecrk-V.5* mutants infiltrated with 1×10^5^ cfu.ml^−1^ *Pst* DC3000 (*Pst*). Data represent average ±SD. Means were not significantly different between WT and mutants when evaluated by a *t*-test (P<0.01, n = 9). dpi, day post inoculation. Experiments were repeated 3 times with similar results.
(TIF)

Figure S3 **Stomatal aperture in** ***lecrk-V.5-2*** **mutant and overexpression lines after bacterial inoculation.** (A) Stomatal aperture of WT Col-0 and *lecrk-V.5-2* after a 1 hr and 3 hr incubation time with MES buffer (Control) or 1×10^8^ cfu.ml^−1^ *Pst* DC3000 (*Pst*). (B) Stomatal aperture in WT Col-0 and overexpression lines (OE-1 and OE-2) after 1, 1.5 and 3 hrs incubation in MES buffer (Control) or 1×10^8^ cfu.ml^−1^ *Pst* DC3000 (*Pst*). Results are shown as mean of ≥60 stomata ± SE. Asterisks indicates significant differences between WT and mutant/OE based on a *t* test (*P*<0.001). All experiments were repeated at least three times with similar results. hpi, hour post inoculation.
(TIF)

Figure S4 **Altered PAMP-induced stomatal closure in lines overexpressing** ***LecRK-V.5*** **.** The stomatal response of lines overexpressing *LecRK-V.5* (OE-1 and OE-2) to different concentrations of flg22 (A), elf26 (B) and LPS (C). Results are shown as mean of ≥60 stomata ± SE. Asterisks indicate significant differences between WT Col-0 and OE lines based on a *t* test (*P*<0.001). All experiments were repeated at least three times with similar results.
(TIF)

Figure S5 **ROS production upon PAMPs treatments.** ROS detected by H_2_DCFDA fluorescence in guard cells of WT Ler and *lecrk-V.5-1* mutant after treatments with MES buffer (Control), 5 µM flg22, 5 µM elf26 or 100 ng.µL^−1^ LPS. Results are shown as mean ± SE. Asterisks indicate significant differences to WT control based on a *t* test analysis (n≥60; *P*<0.001). Experiment was repeated at least three times with similar results.
(TIF)

Figure S6 **Lines overexpressing** ***LecRK-V.5*** **demonstrate a WT stomatal response to MeJA and SA.** Effect of MeJA (A) and SA (B) on stomatal aperture in WT Col-0 and overexpression lines OE-1 and OE-2. Results are shown as mean of ≥60 stomata ± SE. No significant differences between Col-0 and OE lines were observed based on a *t* test (*P*<0.001). All experiments were repeated at least three times with similar results.
(TIF)
