# Supplementary figures and images for: The Arabidopsis Lectin Receptor Kinase LecRK-V.5 Represses Stomatal Immunity Induced by Pseudomonas syringae pv. tomato DC3000
Source: PLoS Pathog. 2012 Feb 9;8(2):e1002513. doi: 10.1371/journal.ppat.1002513 (PMC3276567; doi:10.1371/journal.ppat.1002513)

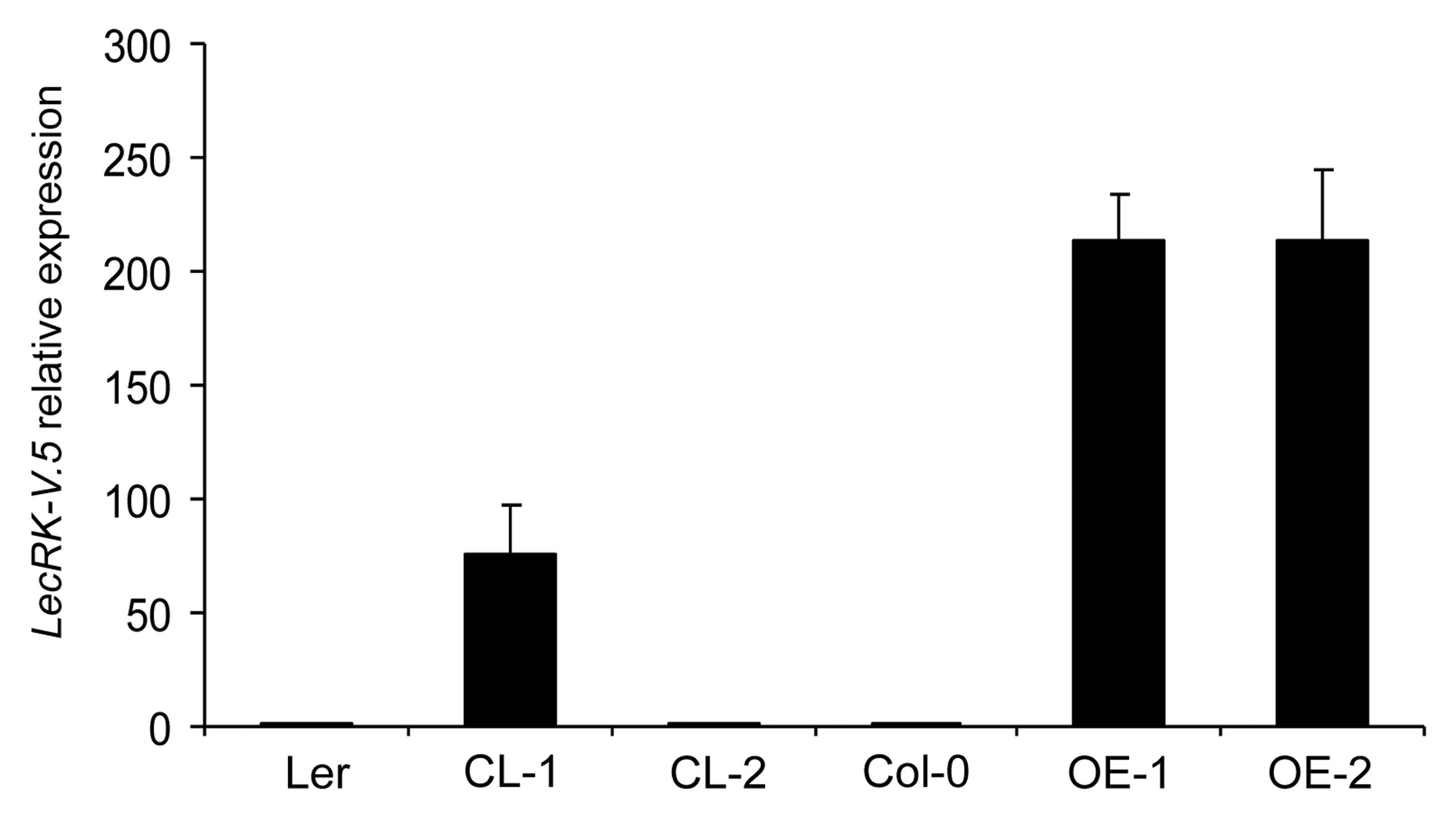

Supplement: Figure S1 — LecRK-V.5 expression levels in transgenic lines. Relative expression levels in WT (Ler) and two complemented lines (CL-1 and CL-2) and WT (Col-0) and two overexpression lines (OE-1 and OE-2). Transcript levels were determined by qRT-PCR and normalized to both EF-1 and UBQ10. Expression levels were compared to WT controls with a defined expression value of 1. Bars indicate SD (n = 6). Experiments were repeated 3 times with similar results. (TIF) [file ppat.1002513.s001.tif]

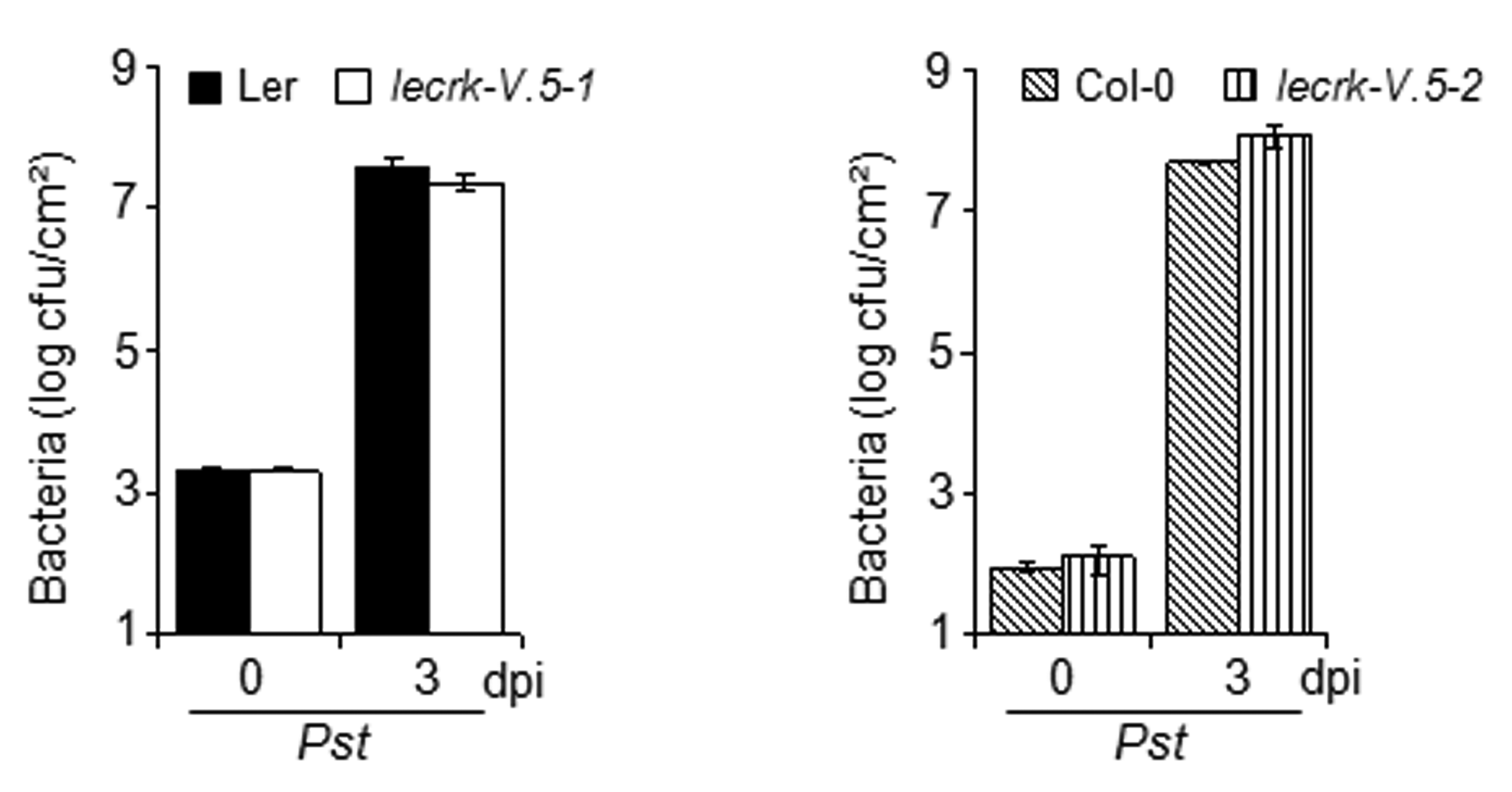

Supplement: Figure S2 — Susceptibility of lecrk-V.5 mutants to Pst DC3000 infiltration-inoculation. Bacterial growth (colony forming units (cfu) per cm leaf area) was determined in Ler, Col-0 and lecrk-V.5 mutants infiltrated with 1×10^5^ cfu.ml−1 Pst DC3000 (Pst). Data represent average ±SD. Means were not significantly different between WT and mutants when evaluated by a t-test (P<0.01, n = 9). dpi, day post inoculation. Experiments were repeated 3 times with similar results. (TIF) [file ppat.1002513.s002.tif]

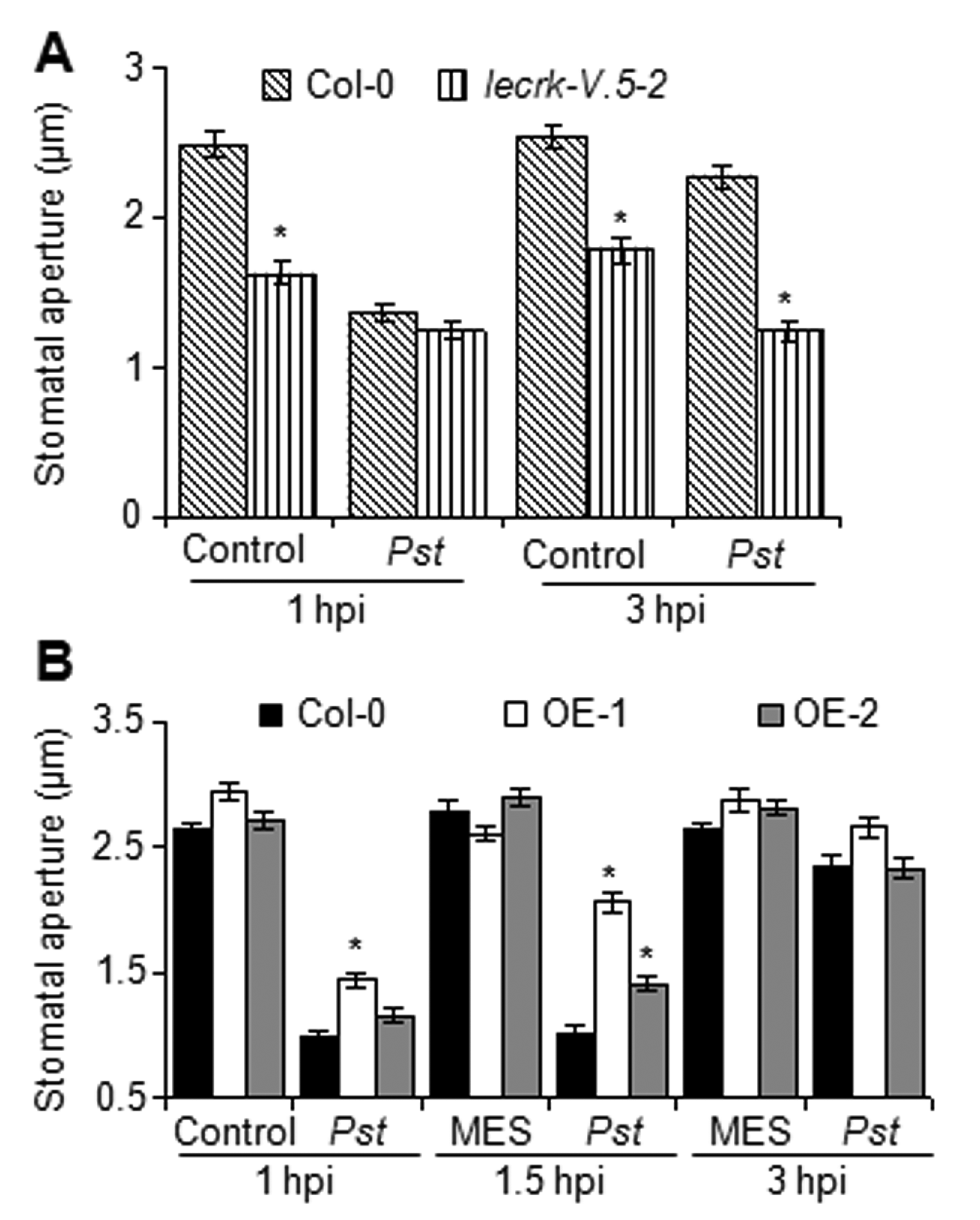

Supplement: Figure S3 — Stomatal aperture in lecrk-V.5-2 mutant and overexpression lines after bacterial inoculation. (A) Stomatal aperture of WT Col-0 and lecrk-V.5-2 after a 1 hr and 3 hr incubation time with MES buffer (Control) or 1×10^8^ cfu.ml−1 Pst DC3000 (Pst). (B) Stomatal aperture in WT Col-0 and overexpression lines (OE-1 and OE-2) after 1, 1.5 and 3 hrs incubation in MES buffer (Control) or 1×10^8^ cfu.ml−1 Pst DC3000 (Pst). Results are shown as mean of ≥60 stomata ± SE. Asterisks indicates significant differences between WT and mutant/OE based on a t test (P<0.001). All experiments were repeated at least three times with similar results. hpi, hour post inoculation. (TIF) [file ppat.1002513.s003.tif]

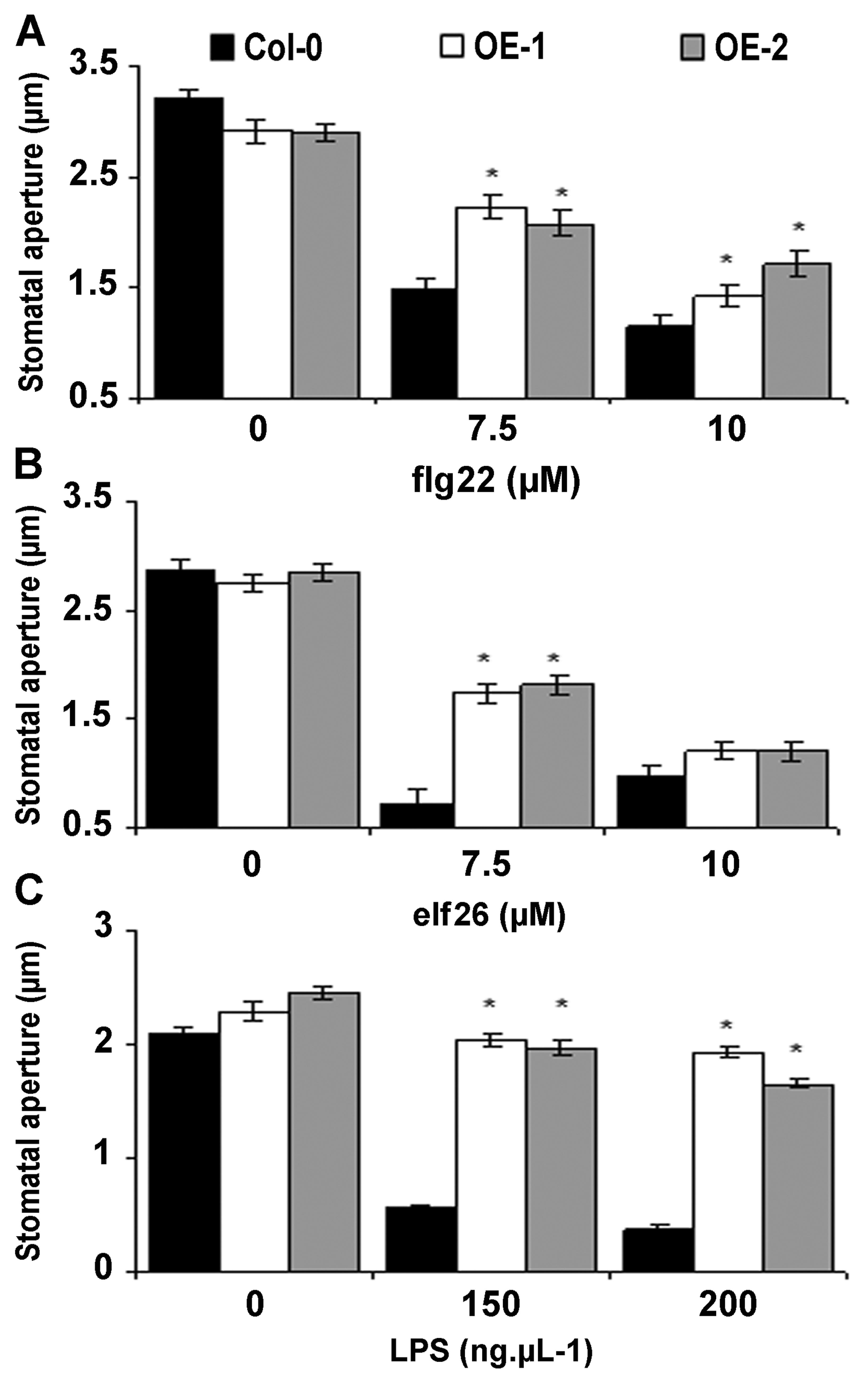

Supplement: Figure S4 — Altered PAMP-induced stomatal closure in lines overexpressing LecRK-V.5 . The stomatal response of lines overexpressing LecRK-V.5 (OE-1 and OE-2) to different concentrations of flg22 (A), elf26 (B) and LPS (C). Results are shown as mean of ≥60 stomata ± SE. Asterisks indicate significant differences between WT Col-0 and OE lines based on a t test (P<0.001). All experiments were repeated at least three times with similar results. (TIF) [file ppat.1002513.s004.tif]

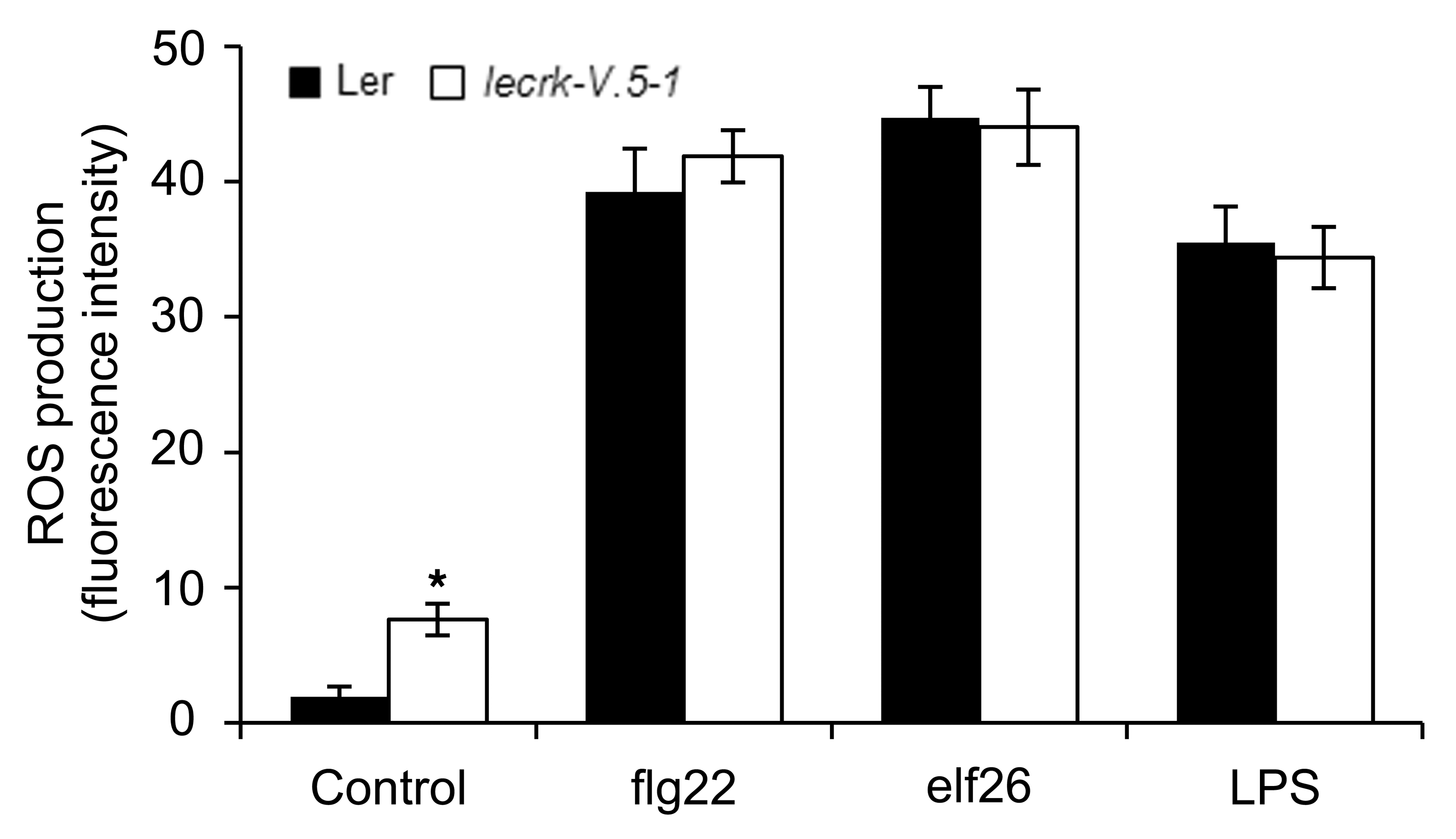

Supplement: Figure S5 — ROS production upon PAMPs treatments. ROS detected by H2DCFDA fluorescence in guard cells of WT Ler and lecrk-V.5-1 mutant after treatments with MES buffer (Control), 5 µM flg22, 5 µM elf26 or 100 ng.µL−1 LPS. Results are shown as mean ± SE. Asterisks indicate significant differences to WT control based on a t test analysis (n≥60; P<0.001). Experiment was repeated at least three times with similar results. (TIF) [file ppat.1002513.s005.tif]

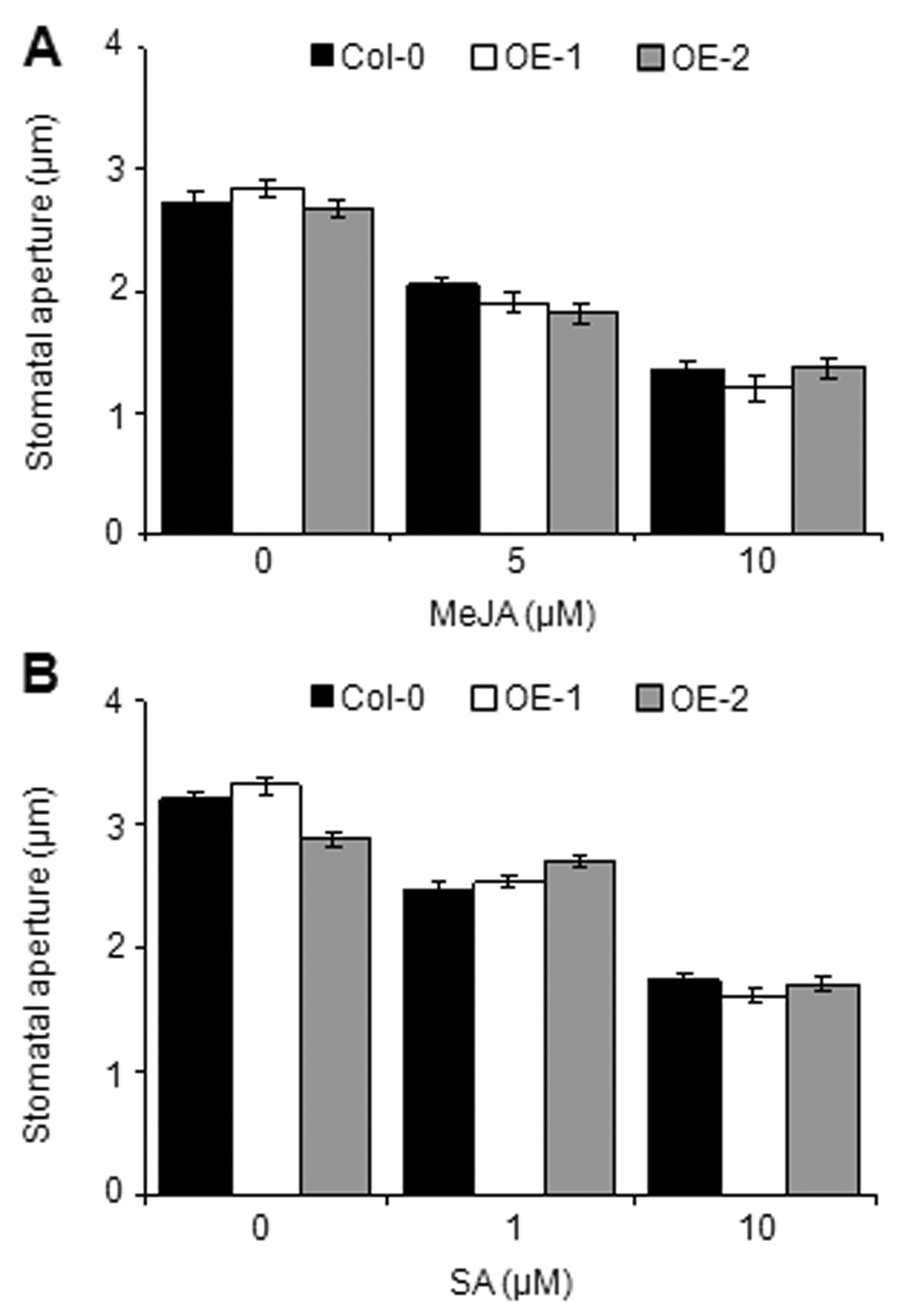

Supplement: Figure S6 — Lines overexpressing LecRK-V.5 demonstrate a WT stomatal response to MeJA and SA. Effect of MeJA (A) and SA (B) on stomatal aperture in WT Col-0 and overexpression lines OE-1 and OE-2. Results are shown as mean of ≥60 stomata ± SE. No significant differences between Col-0 and OE lines were observed based on a t test (P<0.001). All experiments were repeated at least three times with similar results. (TIF) [file ppat.1002513.s006.tif]
